# Comparison of clean intermittent and transurethral indwelling catheterization for the treatment of overt urinary retention after vaginal delivery: a multicentre randomized controlled clinical trial

**DOI:** 10.1007/s00192-017-3452-y

**Published:** 2017-08-30

**Authors:** Femke E. M. Mulder, Robert A. Hakvoort, Jan P. de Bruin, Joris A. M. van der Post, Jan-Paul W. R. Roovers

**Affiliations:** 1 Department of Obstetrics and Gynaecology, Academic Medical Center, Meibergdreef 9 – room H4.240, 1105 AZ Amsterdam, The Netherlands; 2 Department of Obstetrics and Gynaecology, Martini Ziekenhuis, Groningen, The Netherlands; 3 Department of Obstetrics and Gynaecology, Jeroen Bosch Ziekenhuis, Den Bosch, The Netherlands

**Keywords:** Postpartum urinary retention, Clean intermittent catheterization, Transurethral indwelling catheterization, Randomized controlled trial, Micturition symptoms, Voiding dysfunction

## Abstract

**Introduction and hypothesis:**

Overt postpartum urinary retention (PUR) is the inability to void after delivery and affects up to 7% of patients. Clean intermittent catheterization (CIC) and transurethral indwelling catheterization (TIC) are both standard treatments, but have not previously been compared. Clinical guidelines on postpartum bladder management are lacking.

**Methods:**

A total of 85 patients were randomised for TIC (n=45) and CIC (n=40). In total 68 patients (34 patients with TIC and 34 patients with CIC) completed the UDI-6 questionnaire 3 months after delivery.. Patients allocated to TIC received an indwelling catheter for 24 h and if necessary, another catheter for 48 h. Patients with CIC were intermittently catheterized or taught to self-catheterize until adequate voiding with a postvoid residual volume (PVRV) of <150 mL was achieved. The primary outcome was the presence of bothersome micturition symptoms as measured using the Dutch-validated Urogenital Distress Inventory (UDI-6).

**Results:**

Only seven patients (10%) reported bothersome micturition problems 3 months after delivery. No significant differences in the occurrence of micturition symptoms were found. Median PVRV was 800 mL in the CIC group and 650 mL in the TIC group. PVRV was ≥1,000 mL in 24% of the patients. The median duration of catheterization was significantly shorter in the CIC group than in the TIC group (12 h vs. 24 h, *p* < 0,01). In patients with CIC, 35% required only one catheterization before complete bladder emptying occurred. The duration of treatment was not related to the initial PVRV. Both treatments were equally well accepted by the patients.

**Conclusions:**

In patients with overt PUR, CIC is the preferred treatment as a considerable percentage of patients appear to be over-treated when the standard duration of TIC is 24 h. The occurrence of micturition symptoms is not associated with the catheterization method used. CIC is well tolerated in patients with overt PUR.

## Introduction

Postpartum urinary retention (PUR) is the inability to void spontaneously or adequately after delivery. It is a frequently occurring condition in the puerperium. In previous studies the percentages of affected women have varied from 12% to 45% [1–3]. Women experiencing PUR can be subdivided into two clinically different groups; those who experience overt (symptomatic) PUR, i.e. the absolute inability to void spontaneously within several hours of delivery, and those with covert (asymptomatic) PUR who are able to void spontaneously but have increased postvoid residual volumes (PVRVs). Because of the overt nature of the problem, patients with symptomatic PUR are generally identified early, either because micturition is impossible or because of complaints such as abdominal pain or postpartum haemorrhage. Patients with covert PUR are more difficult to identify because spontaneous micturition does occur but the residual volume is high. In case of overt PUR, catheterization is considered to be required because bladder overdistension can potentially result in long-term problems including reduced detrusor contractility, urinary tract infections, hydronephrosis and even kidney failure [4, 5].

With these potentially serious consequences, it is surprising that guidelines for the treatment of PUR are lacking. More than a decade ago the absence of evidence-based protocols on bladder care in the puerperium had already been addressed [6]. However, until now no studies have been published that deal with the optimization of treatment for this problem [6]. Consequently, currently available recommendations regarding treatment of PUR are still not generally evidence-based [7–9]. This absence of clarity results is an undesirable variation in the management of overt PUR among different institutions [10–12]. In many departments, patients are still treated by standard prolonged transurethral indwelling catheterization (TIC) for periods of up to 72 h. This is surprising as it is well known that TIC can cause significant discomfort and the risk of urinary tract infection increases significantly with every day of continuing catheterization [13–15]. Moreover, in different categories of patients with urinary retention, evidence is available that clean intermittent catheterization (CIC) results in earlier normalization of micturition without residual than TIC [16–18].

Whether this is also true in women with PUR after delivery is unknown. A randomized controlled trial was designed with the following aims: to safely prevent bladder overdistension in patients with PUR and concurrently reduce the duration of catheterization and to evaluate whether CIC is associated with less-severe micturition symptoms 3 months after delivery as compared with TIC.

## Materials and methods

Between January 2011 and June 2016 we performed a randomized controlled clinical in five teaching hospitals in The Netherlands. The study was approved by the Medical Ethics Committee of the Academic Medical Centre in Amsterdam, The Netherlands (MEC AMC 10/187). Local approval was obtained in all participating centres. The trial was registered with the Dutch Trial Registry (NTR 2806). During the study period, women aged 18 years and older who were unable to void spontaneously within 6 h of vaginal delivery were asked to participate. After obtaining informed consent, patients were randomized to either TIC for 24 h or CIC.

### Catheterization protocol

In patients allocated to TIC, the catheter was removed after 24 h. If spontaneous voiding occurred, the PVRV was measured using a Bladderscan® (BVI 9400; Verathon, IJsselstein, the Netherlands). If PVRV was less than 150 mL, no further treatment followed. If PVRV remained above 150 mL or if no spontaneous voiding occurred, an indwelling catheter was again inserted and left in place for an additional 48 h. After removal of this indwelling catheter the residual volume was measured again. If micturition was still not possible or if an abnormal PVRV persisted, the patient was instructed in how to perform CIC.

In patients allocated to CIC the frequency of catheterization depended on the residual bladder volume, i.e. two times a day for PVRV ≥200 mL, three times a day for PVRV ≥300 mL and four times a day for PVRV ≥400 mL. As long as residual volumes remained above 150 mL, patients underwent repeated CIC or were instructed in how to perform CIC themselves. Patients who were discharged from the hospital and still required CIC were asked to complete a catheterization diary at home to measure the frequency and duration of CIC and PVRV.

### Urinary culture

When catheterization, either CIC or TIC, was finished, a urine sample was sent to the laboratory for culture. During treatment, investigators were not informed about the results of this culture, and this culture was not part of routine clinical practice. By definition, cultures were regarded as positive when more than 10^5^ colony-forming units were found.

### Outcomes

The primary outcome was the presence of lower urinary tract symptoms 3 months after delivery. In order to assess micturition-related symptoms, all patients were asked to complete the Dutch-validated short version of the Urogenital Distress Inventory (UDI-6) [19, 20]. The UDI-6 is a six-item validated questionnaire used to identify symptoms associated with lower urinary tract dysfunction. The UDI questionnaires are widely accepted by urogynaecological clinics in The Netherlands. Questionnaires were analysed using a four-point scale resulting in total and domain scores, in which higher scores indicate more severe symptoms [21, 22].

Secondary outcomes were duration of catheterization, bacteriuria and patient preference. To learn more about the patients’ experiences and preferences, a questionnaire was developed. Patients were asked to give their opinion on different statements concerning the treatment received 3 months after delivery. These statements included, for example: “Introduction of the catheter was painful”, “Having urinary retention negatively influenced my postnatal period” and, for the TIC group, “I would have preferred to be randomized to CIC” (and vice versa for the CIC group). The statements could be scored from 1 (totally disagree) to 10 (totally agree). These scores were subsequently categorized into disagree (score 1–4), neutral (score 5 or 6) and agree (score 7–10), and then compared between the CIC and TIC groups.

### Sample size calculation

A difference between the two treatment groups of six points in the Obstructive Micturition domain (of the validated quality-of-life questionnaire) was considered to represent a clinically relevant difference between the groups, based on noninferiority. With a power of 80%, an α level of 0.05, and a standard deviation of 3.75, the calculated sample size necessary was 68 (34 in each group) using a two-sided two-sample *t* test. Assuming a drop-out rate of 15%, we planned to include 86 women in this study.

### Randomization

Computerized randomization tables, stratified in blocks and different sizes per hospital, were used for randomization. Because of the obvious difference between treatments, blinding was not possible.

### Statistical analysis

The data were analysed on an intention-to-treat basis. To evaluate differences between the groups, an unpaired Student’s *t* test for continuous variables and Fisher’s exact test for dichotomous variables were used. The Mann–Whitney *U* test was performed to test nonparametric outcomes for statistical significance. Two-sided significance tests were used throughout. A *p* value of <0.05 was considered to be statistically significant. Statistical analysis was performed using SPSS, version 23.0 (IBM Corp., Armonk, NY).

## Results

**Fig. 1:**
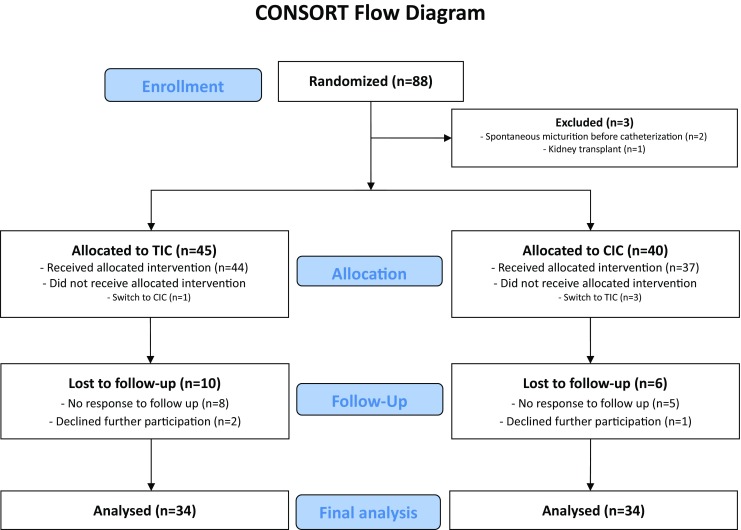
CONSORT flow diagram of the selection of patients for analysis

Data from 85 patients were available for analysis, as shown in Fig. 1. Of these 85 patients, 68 (34 in the TIC group and 34 in the CIC group) completed the UDI-6 questionnaire 3 months after delivery. The data from these patients were used to calculate the primary outcome. Baseline characteristics were not significantly different between the groups (Table 1). The median first urinary bladder volume was 800 mL (range 230–1,600 mL) in the CIC group and 650 mL (range 375–1,800 mL) in the TIC group. Micturition symptoms 3 months after delivery as expressed by the UDI domain scores concerning irritative symptoms, stress symptoms and obstructive symptoms did not significantly differ, as shown in Table 2.

**Table 1:** Patient characteristics

Characteristic	CIC group (*n* = 40)	TIC group (*n* = 45)	*p* value
Nulliparous, %	73	76	NS
Gestational age, mean	39.9	39.7	NS
Maternal age (years), mean	30.7	30.3	NS
Body mass index (kg/m^2^), median	23	23	NS
Duration first stage (h), median	7.5	8.0	NS
Duration second stage (minutes), median	50	38	NS
Induction of labour, %	53	60	NS
Spontaneous vaginal delivery, %	63	70	NS
Assisted vaginal delivery, %	35	29	NS
Birth Weight (g), mean	3,568	3,552	NS
Epidural analgesia, % of total patients	53	51	NS
Episiotomy, %	50	44	NS
Catheterization during labour, %	65	52	NS
First catheterized residual volume (mL), mean	825	796	NS

*NS* non-significant, *CIC* clean intermittent catheterization, *TIC* transurethral indwelling catheterization

**Table 2:** UDI-6 questionnaire scores 3 months after delivery

	CIC group (*n* = 34)		TIC group (*n* = 34)		*p* value
	Mean	Standard deviation	Mean	Standard deviation	
UDI-6 total score	10.9	14.2	8.3	10.0	0.42
Irritative symptoms	11.1	17.9	9.09	13.3	0.63
Stress symptoms	12.4	19.4	9.6	15.6	0.55
Obstructive symptoms	9.3	19.8	6.1	10.1	0.45

*CIC* clean intermittent catheterization, *TIC* transurethral indwelling catheterization

Table 3 shows the time to adequate voiding in both groups. The median durations of catheterization were significantly different between the groups (12 h in the CIC group, 24 h in the TIC group; *p* < 0.01). In the CIC group, 35% of the patients (*n* = 13) needed only a single catheterization before regaining adequate voiding. Overall, 78% of patients in the CIC group required a maximum of four catheterizations and had adequate voiding within 1 day. In the TIC group, 84% of the patients (*n* = 37) were able to void adequately after 1 day of catheterization, and 7 patients (16%) required catheterization for another 48 h. After 72 h of catheterization, only one patient in the TIC group needed to continue with CIC for 1 day. The median first catheterized volumes in patients in the TIC group were 600 mL (range 375–1,800 mL) in those requiring one catheter and 1,000 mL (range 450–1500 mL in those requiring a second catheter.

**Table 3:** Resumption of adequate voiding after delivery in the two catheterization groups

Time to resumption of adequate voiding after delivery		CIC group (*n* = 37)	TIC group (*n* = 44)	*p* value
Within 24 h		64.9% (*n* = 24)	–	–
Number of catheterizations	One	35% (*n* = 13)	–	–
	Two	22% (*n* = 8)	–	–
	Three	8% (*n* = 3)	–	–
At 24 h		78.4% (*n* = 29)	84.1% (*n* = 37)	–
At 48 h		94.6% (*n* = 35)	86.4% (*n* = 38)	–
At 72 h		94.6% (*n* = 35)	97.7% (*n* = 43)	–
After ≥72 h		100% (*n* = 37)	100% (*n* = 44)	–
Median duration of catheterization (h)		12 (0–768)	24 (24–72)	0.01

*CIC* clean intermittent catheterization, *TIC* transurethral indwelling catheterization

In total four patients crossed over to the other treatment group. Three patients allocated to CIC received an indwelling catheter. Of these three patients, two received an indwelling catheter because of vulvar oedema and pain, and one experienced prolonged PUR after initially starting with CIC, and was treated with an indwelling catheter for 10 days and performed CIC for 28 days thereafter. The fourth patient requested CIC after having had an indwelling catheter for 24 h instead of receiving another indwelling catheter. Data from these patients were excluded from Table 3.

The questionnaire on experience of the treatment received was completed by 28 patients in the TIC group and 26 patients in the CIC group. The statement “Introduction of the catheter was painful” was responded to by 16 patients in the CIC group and 21 patients in the TIC group. In the CIC group, 3 patients (18%) agreed with the statement, 3 patients (18%) scored neutral, and 10 patients (63%) disagreed. In the TIC group, none of the patients agreed with the statement, 3 of 24 (14%) scored neutral, and 18 of 24 (86%) disagreed, indicating that introduction of the catheter was not painful. These results were not significantly different (*p* = 0.09). The statement “Having urinary retention negatively influenced my postnatal period” was responded to by 18 patients in the CIC group and 21 patients in the TIC group. In the CIC group, 9 patients (50%) agreed with the statement, 1 patient (6%) scored neutral, and 8 patients (44%) disagreed. In the TIC group, 4 patients (19%) agreed with the statement, 2 patients (10%) scored neutral, and 15 patients (71%) disagreed. These results were not significantly different (*p* = 0.12). The final statement “I would have preferred the other (not allocated) treatment” was responded to by 26 patients in the CIC group and 28 patients in the TIC group. In the CIC group, 4 patients (15%) agreed with the statement, and 22 patients (85%) disagreed. In the TIC group, 6 patients (21%) agreed the statement, 4 patients (14%) scored neutral, and 18 patients (64%) disagreed. These results were not significantly different (*p* = 0.09).

## Discussion

In this study, we showed that in postpartum women unable to void spontaneously within 6 h of vaginal delivery, the presence of micturition symptoms and patient preference regarding catheterization method were similar for both regimens 3 months after delivery. Bladder emptying normalized earlier with CIC than with TIC.

The prevention of bladder overdistension is clearly the reason to screen and treat patients unable to void spontaneously. Assuming that these basic requirements are met by both treatments, we wanted to know whether the intermittent filling and emptying of the bladder, which mimics normal physiology, would be beneficial to the function of the bladder in the medium to longer term. Therefore, the primary outcome of this randomized controlled trial was the presence of bothersome micturition symptoms 3 months after vaginal delivery in patients requiring catheterization for overt PUR. Regarding total and domain scores from the UDI-6 questionnaire, no significant differences between the two catheterization methods were observed. This could have been related to the low incidence of micturition symptoms in our population. Only 10% (seven ) of the included patients reported bothersome micturition problems 3 months after delivery (defined as a total UDI-6 score of ≥25 points. This low incidence is comparable to the rates found in previous studies [23–25]. Our study showed that the functionality of the bladder in the postpartum period is hardly affected by temporary (over)distension due to PUR.

In our population, the median residual volume at the moment of catheterization was 700 mL. Of the included patients, 24% had a residual volume of more than 1,000 mL. Acute urinary retention in other situations, such as after (spinal) analgesia or neurological surgery or in relation to prostatic hyperplasia, has been shown to cause bladder overdistension that can lead to chronic impairment of the capacity of the bladder to (adequately) empty [26]. Therefore, our hypothesis was that these patients would have a considerable risk of bladder overdistension, resulting in injury to the detrusor and as a consequence long-term micturition problems [4, 26]. However, the course of PUR, even with the high residual volumes found, appeared to be relatively benign, as PUR resolved after a maximum 72 h in nearly all patients [27, 28]. This implies that after delivery the bladder is not only highly resilient but also that, to a certain extent, residual volumes might possibly be part of normal puerperal physiology.

Our study showed that in patients with symptomatic PUR, CIC is preferred over TIC. Regarding the duration of catheterization, there was a significant difference in favour of CIC, with a median catheterization duration of 12 h compared with 24 h in patients with TIC (*p* < 0.01). Furthermore, 35% of patients with CIC were able to void spontaneously and adequately after only a single catheterization. The duration of catheterization was not correlated with the initial bladder volume at the first catheterization. The advantage of CIC could be associated with the difference in bladder training. By intermittent drainage and filling of the bladder, the bladder is stimulated earlier to identify the difference between “full” and “empty”, leading to spontaneous voiding. In contrast, by placing an indwelling catheter, bladder stimulation is postponed and bladder training delayed. While Lakeman et al. [29] have shown that even in patients who have undergone vaginal colporrhaphy, urinary retention is not related to bladder outlet obstruction, and it is plausible that in patients with overt PUR the disturbance of central inhibition due to pain has a larger role than, for example, periurethral oedema.

In this study CIC as well as TIC were well accepted by the study participants. This is important since in daily clinical practice, nursing and medical staff often believe that CIC after delivery is either too difficult or too bothersome, resulting in frequent use of TIC. In our study, 85% of patients with CIC and 64% of patient with TIC tolerated the allocated treatment well. We have no information on duration of hospital stay due to urinary retention. However, it is plausible that CIC can also be the more cost-effective option. Taking into account that patients consenting to randomization were willing to accept either catheterization method, our results show that CIC is a valid alternative to TIC.

A limitation of this study was the number of missing urine cultures. Although collecting a urine sample from patients who had finished catheterization or had been discharged with CIC was part of the study protocol, data on bacteriuria were only available in 34% of the included patients (10 patients with CIC and 19 patients with TIC). Therefore, we could not compare data on (asymptomatic) bacteriuria between the two treatment groups. However, previous studies have shown that the risk of (asymptomatic) bacteriuria is higher in patients receiving an indwelling catheter than in those undertaking CIC [16, 30]. As catheter-associated bacteriuria is a frequently diagnosed complication, the rationale for the benefits of CIC are interesting. It is known that bacterial infection can progress during catheterization, either directly due to insertion of the catheter or by colonization, as bacteria can ascend from the meatus of the urethra along the catheter (intraluminally and/or extraluminally) [14, 31]. Also spontaneous resolution of bacteriuria has been described when spontaneous micturition, i.e. without a catheter, becomes possible. With CIC, the bladder is emptied so that potentially infected urine does not pool in the bladder, and this is an advantage over TIC in which potentially infected foreign material remains in the bladder causing colonization.

Our results could contribute to the creation of guidelines on the management of the bladder after delivery. Evidence is needed to create protocols on bladder care in the puerperium, but no randomized studies have been published on this topic [6]. Sathiyathasan et al. performed an audit of the cases of 40 randomly selected patients from the postnatal ward, and showed poor compliance with the assessment and documentation of spontaneous micturition within 6 h of delivery [32]. Moreover, in international guidelines on postpartum care, voiding problems after delivery are often not mentioned. One of the few opinion-based recommendations on PUR can be found in the RCOG guidelines on operative vaginal delivery. These suggest that “timing and volume of the first void should be monitored and documented” and that “a post void residual volume should be measured if retention is suspected”. If PUR is present, placement of an indwelling catheter for 12 h is advised [7].

This study showed that there are no differences in longer term bothersome micturition symptoms between patients with CIC and patients with TIC, but the duration of catheterization for symptomatic PUR is significantly shorter in patients with CIC than in those with TIC. Furthermore, the duration of catheterization seems not to be related to the initial residual volume. Although we had insufficient data on cost effectiveness and bacteriuria, we would recommend that future guidelines and protocols for the care of the bladder after delivery include the results of this first randomized clinical trial, and that CIC becomes the standard treatment for symptomatic PUR. If the clinician considers that TIC is necessary, for example if extensive periurethral lacerations are present that would make the introduction of a catheter painful or result in immobility of the patient, the indwelling catheter should be removed after 24 h to evaluate whether spontaneous voiding is possible.

### Conclusions

In patients with symptomatic PUR treated with either CIC or TIC, the prevalence of micturition-related symptoms does not differ between the two treatment methods. Patients with CIC regained spontaneous micturition significantly earlier than patients with TIC (12 vs. 24 h). CIC should therefore be the preferred treatment in patients with symptomatic PUR, regardless of the residual volume. If TIC is considered necessary, the indwelling catheter should be removed after a maximum of 24 h to start bladder training.
